# Bedside Peripheral Ultrasound-Guided Vascular Access in 253 Patients Hospitalized With COVID-19 Pneumonia: A Retrospective Italian Study

**DOI:** 10.7759/cureus.24157

**Published:** 2022-04-15

**Authors:** Francesco Oleari, Chiara Citterio, Salvatore Bontini, Oriella Grassi, Corrado Gozzo, Jessica Premoli, Maria Pia Mezzi, Stefano Roscio, Monica Muroni, Gabriele Cremona, Claudia Biasini, Patrizia Mordenti, Luigi Cavanna

**Affiliations:** 1 Onco-hematology, Hospital Piacenza, Piacenza, ITA

**Keywords:** hospitalized patients, safety, ultrasound-guided, vascular access device, covid-19

## Abstract

Background

Several studies have recommended the use of vascular access in the treatment of COVID-19 patients. However, little is known about the utility and safety of using a peripheral ultrasound-guided vascular access device (UGVAD) at the bedside of hospitalized COVID-19 patients. To examine this, a retrospective monocenter study was carried out at the oncology-hematology department of Azienda Sanitaria di Piacenza, Italy.

Methods

We retrospectively analyzed data from three general hospitals in a district in North Italy on the positioning of UGVADs used with hospitalized COVID-19 patients. The positioning of the VAD was performed by a dedicated team using ultrasound guidance. The primary endpoint was the duration of VAD until the patient’s recovery or death. The secondary endpoints were complications of the use of VADs, which included vein thrombosis, infections, device malfunction, and viral contamination of the operators.

Results

Between February 21, 2020, and April 30, 2020, 253 consecutive hospitalized patients with COVID-19 pneumonia underwent UGVAD positioning. A midline was inserted in 88.53% of the patients, while peripheral central venous catheters and femoral central catheters were inserted in 9.88% and 1.59% of the patients, respectively. The mean lifespan of the VADs was 10.36±9.96 days (range: 1-73). Primary endpoint: The use of the VAD allowed the planned treatment in 92.88% of the patients; in the remaining 7.12%, the VAD was repositioned. Secondary endpoints: Complications of VAD were registered in 15.02% of the patients (dislocation, 9.49%; infection, 1.98%; thrombosis, 1.58%; occlusion, 1.19%; and malfunction, 0.79%). No contamination of the operators was registered.

Discussion and conclusion

With the limitation of being a retrospective study, our report suggests that ultrasound-guided positioning of VAD may allow the safe clinical management (drug infusion, hydration, parenteral nutrition, and phlebotomy) of hospitalized COVID-19 patients. The observance of recommended procedures protected all operators from infection.

## Introduction

In December 2019, a new enveloped RNA beta-coronavirus was identified and named severe acute respiratory syndrome coronavirus 2 (SARS-CoV-2), which causes severe pulmonary disease in 14% of infected people [1,2]. The WHO declared the spread of coronavirus disease 2019 (COVID-19), caused by SARS-CoV-2, a public health emergency of international concern, subsequently pronouncing it to be a pandemic [3]. During the first wave in Italy, the regions most affected by COVID-19 were Lombardy, Emilia Romagna, and Veneto. The city of Piacenza (in the Emilia Romagna region) is located near the epicenter of the outbreak of COVID-19, and the catastrophic nature of the outbreak in Lombardy has been widely publicized [4]. Hospitalized COVID-19 patients often need vascular devices for the delivery of fluids, parenteral nutrition, medication, phlebotomy, and to avoid multiple venipuncture attempts [5,6]. A previous study by the authors reported the results of 1,978 ultrasound-guided central venous catheter (CVC) insertional procedures [7], and the authors previously published the results of a study on ultrasound-guided central venous catheterization for home parenteral nutrition and hydration in 207 advanced-stage cancer patients [8].

In January 2012, a vascular access devices (VADs) team was established by the health authority in the oncology-hematology department at the hospital of Piacenza (North Italy). This team was composed of six trained nurses, an additional nurse in the role of VAD team leader, and a medical doctor, as previously reported [9].

The VAD team initially served the care of cancer patients in the oncology and hematology department of the Piacenza hospital. Subsequently, the activity of the team was extended to non-cancer patients with a need for VADs in the district of Piacenza (290,000 inhabitants), and in the first two years of activity, the team implanted 3,268 vascular devices, 49% of which were peripherally inserted central catheters (PICCs) and 51% were midline [9]. During the COVID-19 pandemic, many patients have been admitted to hospitals in Piacenza, and the VAD team has been engaged in positioning ultrasound-guided vascular access for these patients when necessary. Several papers have included recommendations for the use of vascular access in COVID-19 patients [5,6,10-12]. However, data on the results of positioning vascular devices in COVID-19 patients are fragmentary and poor [13,14]. In this retrospective study, we report the results of consecutive insertional procedures in 253 hospitalized COVID-19-infected patients in different phases of the disease [15,16].

## Materials and methods

All the VADs were placed at the bedside under ultrasound guidance, as previously reported by our group [9], and were either peripheral venous catheters (midline) or central venous catheters (CVC). CVCs were classified as PICCs or femoral inserted central catheters (FICCs). All venous access catheters were positioned according to international recommendations and guidelines [10]. The patients included in this study were diagnosed with laboratory-confirmed SARS-CoV-2 infections, with reverse-transcription polymerase chain reaction found in nasal-pharyngeal swabs. The infection had caused the respiratory illness COVID-19, defined as an oxygen saturation (SaO2) of 94% or less while breathing ambient air or a ratio of the partial pressure of oxygen (PaO2) to the fraction of inspired oxygen (FiO2) of less than 300 mmHg.

The choice of catheter depended on the clinical needs of the patient. Midline catheters were positioned in patients who needed infusion drugs compatible with peripheral veins and a duration of treatment of about two weeks. PICCs were inserted in patients who needed high-flow infusion, hemodynamic monitoring, and infusion for more than two weeks, and FICCs were positioned in emergency situations in critically ill patients for high-flow infusions and hemodynamic monitoring. Jugular access was avoided because the majority of COVID-19 hospitalized patients require non-invasive ventilation devices, such as helmets and masks, or continuous positive airway pressure (CPAP). According to the Guidelines for the Diagnosis and Treatment of Novel Coronavirus-infected Pneumonia [16], COVID-19 patients are clinically classified into four categories: mild, moderate, severe, and critical.

Because the vascular devices were positioned in the COVID-19 ward, the operators used personal protective equipment (PPE) as recommended [17]. To reduce the spread of the virus, a ward-dedicated ultrasound machine was used. The electrocardiography method was applied to assess the right position of the central catheter tip (as previously reported [18]), and all operators wore a mask with protective filter type FFP2 or FFP3 plus a surgical mask, gloves, a full suit, goggles or face shield. Each patient was identified with a unique recognition code, and the following information for each patient was entered into a Microsoft Excel file (Microsoft Office version 2010, Microsoft® Corp., Redmond, WA): age, sex, symptoms, comorbidities, COVID-19 severity, use of oxygen therapy, and type, duration, and complications of the VAD.

The primary endpoint was the duration of the VAD until the patient’s recovery or death. The secondary endpoints were the complications of the VAD, including vein thrombosis, infections, or malfunctioning of the VAD, and contamination of operators.

Statistical analysis

The patients were registered with a unique recognition code in a Microsoft Excel file (Microsoft Office version 2010). Quantitative variables were described using mean and standard deviation (SD), and qualitative variables were described by absolute and percentage frequencies.

This retrospective study was approved by the Local Ethics Committee AVEN (Area Vasta Emilia Nord), number 2020/0079285.

## Results

Between February 21, 2020, and April 30, 2020, 253 patients hospitalized with COVID-19 pneumonia underwent vascular device positioning. The clinical data are reported in Table 1.

**Table 1:** Demographics and clinical characteristics of 253 patients hospitalized with COVID-19 pneumonia COPD: chronic obstructive pulmonary disease, CVC: central venous catheter, SD: standard deviation, PICC: peripherally inserted central catheter

Characteristic	Patients, n=253
Sex	
Female n (%)	94 (37.15)
Male n (%)	159 (62.85)
Age (mean±sd) (range)	71.17±11.84 (29-98)
COVID-19 acute pneumonia n (%)	253 (100)
Severity of COVID-19	
Moderate n (%)	115 (45.45)
Severe n (%)	132 (52.17)
Critical n (%)	6 (2.37)
Copathologies	
Obesity n (%)	42 (16.60)
Hypertension n (%)	145 (57.31)
COPD n (%)	13 (5.14)
Cancer n (%)	60 (23.72)
Chronic inflammatory disease n (%)	10 (3.95)
Neurologic disease n (%)	57 (22.53)
Cardiovascular disease n (%)	85 (33.6)
Diabetes n (%)	65 (25.69)
Type of vascular access	
Midline n (%)	224 (88.53)
PICC n (%)	25 (9.88)
Femoral CVC n (%)	4 (1.59)
Device duration (mean±sd)	10.36±9.96
Oxygen treatment n (%)	250 (98.81)
Follow up	
Deceased n (%)	90 (35.57)
Improved and discharged n (%)	163 (64.43)

The mean age was 71.17 years (range 29-98), and 159 (62.85%) were male. The majority of the patients had one or more comorbidities: 57.31% had hypertension, 33.6% had coronary disease, 25.69% had diabetes, and 23.72% had cancer. All the patients had COVID-19 pneumonia: 45.45% had moderate, 52.17% had severe, and 2.37% had critical COVID-19 pneumonia [10].

A VAD was inserted under ultrasound guidance for each patient. Midline was the VAD type most often used, inserted in 88.53% of the patients. A PICC was inserted in 9.88% of the patients, and a FICC was inserted in the remaining 1.59%. The mean lifespan of the VADs was 10.36±9.96 days (range 1-73).

The majority of patients (98.81%) who underwent VAD positioning were in oxygen therapy.

Primary endpoint

The VAD allowed for the planned treatments in 92.88% of the patients, while it was necessary to reposition it in the remaining 7.12%. The follow-up of the patients showed that 90 (35.57%) deceased while 163 (64.43%) were discharged.

Secondary endpoints

Complications related to the VAD were reported in 38 out of 253 patients (15.02%). The most common complication was dislocation, registered in 24 out of 253 patients (9.49%), while infection, thrombosis, occlusion, and malfunction were reported in five out of 253 (1.98%), four out of 253 (1.58%), three out of 253 (1.19%), and two out of 253 (0.79%) patients, respectively (Table 2).

**Table 2:** Results of ultrasound-guided peripheral vascular access device insertion in patients hospitalized with COVID-19 based on primary and secondary endpoints VAD: vascular access devices

Primary endpoint	
VAD allowed the planned treatment n (%)	235 (92.88)
VAD repositioned n (%)	18 (7.12)
Secondary endpoints	
VAD complications n (%)	38 (15.02)
Dislocation n (%)	24 (9.49)
Infection n (%)	5 (1.98)
Thrombosis n (%)	4 (1.58)
Occlusion n (%)	3 (1.19)
Malfunction n (%)	2 (0.79)
Operator contamination n (%)	0 (0)

No contamination of the operator was reported.

## Discussion

The majority of patients hospitalized with COVID-19 will require a VAD for drug administration, hydration, and nutrition. In addition, the majority of these patients will need invasive or non-invasive ventilation, such as that provided by a helmet or mask, making the administration of oral or enteral hydration or nutrition difficult; in these cases, VADs are essential.

Many studies have made recommendations on the type of positioning and management of VADs in COVID-19 patients [5,6,10-12]. However, data on the efficacy and safety of VAD positioning in hospitalized COVID-19 patients are fragmentary and poor [13,14]. In this study, 253 patients hospitalized with COVID-19 underwent VAD positioning. The majority of the VADs inserted were midlines, at 88%. The midline VAD allowed for both the taking of blood samples and giving infusions, and these VADs were repositioned in only 7.12% of cases. It is well known that SARS-CoV-2 promotes a hypercoagulative state in infected patients. Recently, Sebolt et al. [13] performed a retrospective multi-center cohort study of patients hospitalized with COVID-19 who received VADs (midline, PICC, non-tunneled CVC, hemodialysis catheter, or port catheter); 261 COVID-19 patients from 40 hospitals were included in this study. The authors reported a higher rate of venous thromboembolism (VTE) (10.0%) in patients who underwent VAD positioning than in those who did not. In our study, the incidence of VTE was very low (1.58%). However, it must be emphasized that almost all the patients included in our study were already being treated with enoxaparin for underlying disease when the VAD was positioned, and this treatment could have reduced the incidence of VTE.

During the first and successive waves of the COVID-19 pandemic, the hospitals in North Italy faced a dramatic and unfamiliar situation, and almost all the doctors and nurses were working on the treatment of patients with SARS-CoV-2 infection. Fortunately, the vascular access team of our hospital was kept intact and could respond quickly to the intravenous needs of hospitalized COVID-19 patients. In this study, we found that the use of electrocardiogram guidance technology for verification of peripherally inserted central catheter tip placement in the lower third of the superior vena cava at the junction with the right atrium was very useful, as well as being cheaper and quicker than performing a post-procedural chest X-ray [18]. This mode was initiated by the VAD-team in 2017.

Our study has some limitations. First, since this was a retrospective study, our results should be viewed with appropriate caution. Second, the number of patients in our study was small when compared to the thousands of patients hospitalized with COVID-19 worldwide. Despite these limitations, our findings are among the first on the utility and safety of ultrasound-guided insertion of vascular devices in hospitalized COVID-19 patients.

## Conclusions

To the best of our knowledge, our study is novel. Our results suggest that vascular devices allow the safe clinical management of drug infusion, hydration, parenteral nutrition, and phlebotomy of patients hospitalized with COVID-19 infection and that VADs can be safely inserted and easily replaced when necessary by a dedicated team using ultrasound guidance.
